# Heart transplant in a patient with acute onset of heart failure and massive bi-ventricular thrombi: A case report

**DOI:** 10.1016/j.jccase.2025.05.003

**Published:** 2025-06-06

**Authors:** Shaden Daloub, Rhythm Vasudeva, Amandeep Goyal, Emily Newton, Hirak Shah, Matthew Danter, Tyler Zorn, Timothy Fields, Tarun Dalia

**Affiliations:** a Department of Cardiovascular Medicine, Kansas University Medical Center, Kansas, KS, USA; b Cardiothoracic Surgery Department, Kansas University Medical Center, Kansas, KS, USA; c Pathology Department, Kansas University Medical Center, Kansas, KS, USA

**Keywords:** Biventricular thrombi, Severe heart failure, Pulmonary embolism, Heart transplant, Veno-arterial extracorporeal membrane oxygenation

## Abstract

Massive biventricular thrombi are a rare but serious complication of acute heart failure with reduced ejection fraction, presenting significant challenges in management. These thrombi can cause coronary thrombi leading to hemodynamic instability and raise the risk of systemic embolism. A 42-year-old male with a past medical history of type 2 diabetes mellitus presented with cardiogenic shock and pulmonary embolism. He was found to have new onset of heart failure with left ventricular ejection fraction of 15 % and harboring large biventricular thrombi. Stress test showed over 50 % of his myocardium was infarcted. Due to these findings, after a multidisciplinary team discussion, he was placed on veno-arterial extracorporeal membrane oxygenation (VA-ECMO) as bridge to orthotopic heart transplant (OHT), for hemodynamic support and prevention of distal embolization. He subsequently underwent OHT as an INTERMACS category 1 a few days later. This rare and complicated case highlights the importance of a multidisciplinary team approach, and utilization of VA-ECMO in an end-stage cardiomyopathy patient with large biventricular thrombi as bridge to OHT.

**Learning objective:**

Veno-arterial extracorporeal membrane oxygenation can be utilized to prevent systemic embolization as a bridge to orthotopic heart transplant in patients with biventricular thrombus and end-stage cardiomyopathy.

Heart transplant can be a lifesaving treatment in a patient with biventricular thrombi and extensive non-viable myocardium.

## Introduction

Ventricular thrombus formation is a rare but dreadful complication associated with severe systolic dysfunction - specifically when the ejection fraction (EF) is <35 % [1]. The incidence of left ventricular (LV) thrombus in heart failure is around 6.1 % [2].

The pathophysiology of LV thrombus formation relates to factors associated with Virchow's triad - endothelial injury, hypercoagulability, and stasis of blood flow [3]. Blood stasis results from ventricular dysfunction which can lead to the development of intramural thrombi [1]. Hypercoagulable states, such as hypereosinophilic syndrome and antiphospholipid antibody syndrome [4] are established risk factors for biventricular thrombi. However, biventricular thrombi formation has also been documented in association with, substance-induced cardiomyopathy, myocarditis, peripartum cardiomyopathy, heart failure with reduced ejection fraction, and myocardial infarction [5]. However, the occurrence of biventricular thrombi is particularly rare.

Management of ventricular thrombus remains a complex issue. There are no clear guidelines available for management of patients with biventricular thrombus from major cardiology organizations such as the American Heart Association, American Stroke Association, and American College of Cardiology [6].

In this report, we explored the presentation and management of a male patient with acute severe heart failure complicated by massive biventricular thrombi who underwent a successful cardiac transplantation.

## Case report

A 42-year-old male with a past medical history of hypertension, type 2 diabetes mellitus, and hyperlipidemia presented with shortness of breath on exertion, progressive lower limb swelling extending to his knees, and loss of appetite. He reported previous high alcohol consumption for six years prior to quitting four years ago; however, he denied smoking or any illicit drug use. The patient also denied any family history of heart attacks, heart failure, or sudden cardiac deaths. His symptoms began a month before presentation including coughing and shortness of breath. He denied any chest pain, palpitation, near syncope, or syncope. He denied any prior COVID positive result. During the physical examination, the patient appeared dyspneic and anxious. He had jugular venous distention. His bilateral lower extremities showed 3+ edema, petechiae, and superficial wounds. Lung examination revealed decreased bilateral air entry in the lower lung zones. Cardiac examination revealed normal S1 and S2, tachycardic with heart rate 118 beat per minute, and a blood pressure of 110/90 mmHg.

**Fig. 1:**
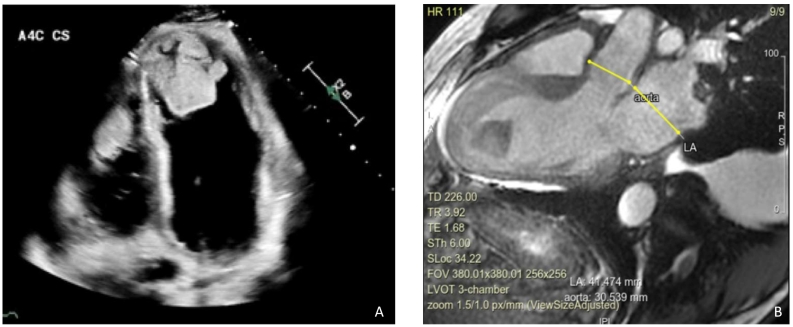
**(**A) Transthoracic echocardiography showing a severely dilated left ventricle with biventricular thrombi. **(B)** Cardiac magnetic resonance imaging shows scars in both right ventricle and left ventricle with nonviable myometrium in half of the left ventricle.

**Fig. 2:**
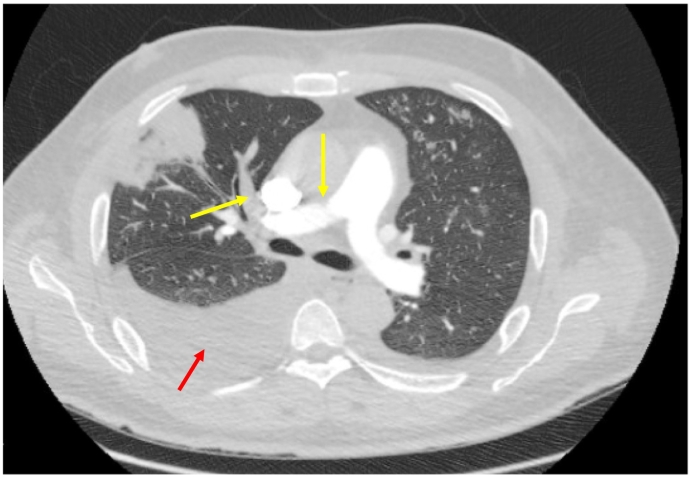
Computed tomography angiography of the chest showing pulmonary embolism with yellow arrows pointing to the pulmonary embolism in the right pulmonary artery and the red arrow pointing to the right pleural effusion.

Initial laboratory work-up displayed a red blood cell count of 5.58 10*6/uL, white blood cell count of 12.4 10*3/uL, platelet count of 262 10*3/uL, prothrombin time of 16.5 (9.5–14.2) seconds, international normalized ratio (INR) of 1.5 (0.8–1.2), creatinine of 1.47 mg/dL, albumin of 3 g/dL, protein of 6.2, lactic acid of 5.3 (0–2) mmol/L, N-terminal pro-B-type natriuretic peptide of 10,000 pg/mL (<125 pg/ml), D-dimer of 27,000 ng/mL (<500), anti-thrombin of 67 % (80–120 %), hexagonal anticoagulant of 23 (0−11), protein C of 51 % (70–130 %), aspartate aminotransferase of 60 U/L, alanine aminotransferase of 68 U/L, alkaline phosphatase of 555 U/L, and a HbA1c of 7.9 %. Factor V Leiden was negative, cardiolipin immunoglobulin (Ig) G and IgM were normal. Baseline activated clotting time (ACT), activating clotting time, point-of-care testing of ACT by iSTAT (Abbott, Abbott Park, IL, USA) and dilute russel venom results were all normal. Genetic mutations for hypercoagulable conditions such as JAK2, protein C, and antithrombin deficiency were negative, COVID test was negative. The hypercoagulable work-up including cancer work-up and laboratory results performed with guidance from our hematology colleagues was unremarkable. Transthoracic echocardiography (TTE) **(**Fig. 1A**)** revealed a severely dilated LV with severe systolic dysfunction, severe diffuse hypokinesis with LV apical akinesis, and an estimated LVEF of 10–15 %. There was moderate right ventricular (RV) dilatation with reduced RV systolic function. Massive, multilobar thrombi were also noted in the RV and LV apices. The RV thrombi precluded from proceeding with right heart catheterization to measure the hemodynamics to avoid dislodgement of these thrombi and causing more pulmonary embolism. A stress positron emission tomography (PET) scan was performed that showed a large area of infarction involving the anterior wall, entire inferior wall, and distal anterolateral wall of the LV. There was no ischemia identified and myocardial blood flow analysis showed severely reduced peak stress and resting myocardial blood flow. A computed tomography angiography of the chest revealed multiple segmental and subsegmental pulmonary emboli in both lungs including several occlusive filling defects which correlated with areas of lung infarcts predominantly involving the anterior right upper, lateral right middle, and anterior basal right lower lobes as well as a moderate right-sided pleural effusion **(**Fig. 2**).** Ultrasound of the abdomen showed hepatomegaly, dilated veins, and trace ascites, suggestive of fluid overload. Bilateral lower extremity venous ultrasound results were negative for deep venous thrombosis. An arterial ultrasound of the lower extremity showed occlusion of the right common femoral artery. Cardiac magnetic resonance imaging (MRI) showed his LVEF of 15 %, RVEF of 15 %–20 %, as well as mid to distal scars in both RV and LV with nonviable myocardium in half of the LV. The cardiac MRI also showed scarring of similar myocardial segments as described in the results of stress PET scan **(**Fig. 1B**).**

Given the patient's presentation of cardiogenic shock and newly discovered severely reduced EF with thrombus in both ventricles, the patient was transferred to the cardiac intensive care unit the next day. Gastrointestinal team consultation also ruled out stomach cancer as a cause of the embolism after performing an esophagogastroduodenoscopy.

Furosemide and heparin drip were initiated. Owing to the significant non-viable myocardium on cardiac imaging, the chances of myocardial recovery were thought to be minimal, and the patient was considered to have end-stage cardiomyopathy. Given his critical condition and instability, adding guideline-directed medical therapy was not an option. An urgent multidisciplinary heart failure transplant committee was convened, and a decision was made to utilize veno-arterial extracorporeal membrane oxygenation (VA-ECMO) to prevent systemic embolization and as a bridge to orthotopic heart transplantation (OHT). He was listed as UNOS (United Network of Organ Sharing) status 1 by exception after committee discussion.

**Fig. 3:**
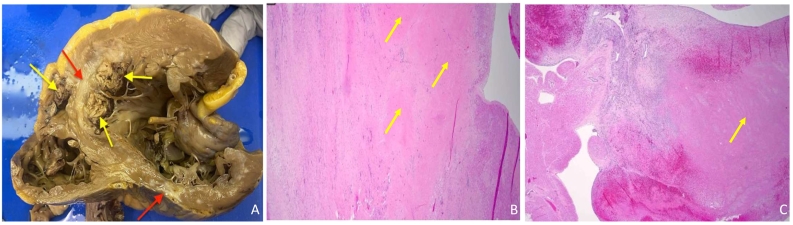
**(A)** Gross pathology for the heart with yellow arrows pointing to thrombi and red arrows pointing to scars. **(B)** Microscopic pictures for left ventricle with yellow arrows pointing to scars. **(C**) Microscopic picture for right ventricle thrombus with yellow arrow pointing to scars.

Four days after being placed on VA-ECMO, the patient underwent an OHT. Intra-operative transesophageal echocardiogram at the time of heart transplant showed similar size of thrombi in both ventricles as was noted in the echocardiogram done on admission. The explanted heart pathology showed a left atrial thrombus consistent with recent thrombus formation, extensive thrombosis in both RV and LV **(**Fig. 3**),** calcific atherosclerosis of coronary arteries, and extensive scarring in anterior septum and posterior LV with patchy repair in the RV and LV adjacent to scarring; this picture was consistent with ischemic cardiomyopathy.

Six days after the transplantation the patient developed RV dysfunction, requiring inotropic support, and a pericardial effusion. Endomyocardial biopsy showed ischemic injury and mixed infiltrate suggesting acute cellular rejection (grade 2R). He was given high-dose steroids (methylprednisolone 500 mg × 3 days) followed by prolonged steroid taper to treat rejection. For transplant immunosuppression, tacrolimus with a goal of 10–12 ng/ml, mycophenolate 720 mg twice daily and prednisone were utilized. We were then able to wean the patient off the inotropes and a repeat biopsy showed resolution of acute rejection.

After 20 days from heart transplantation, the patient was discharged home on anticoagulation with direct oral anticoagulants due to the presence of pulmonary embolism. He will require close follow-up to monitor for transplant rejection and other complications.

## Discussion

To the best of our knowledge, very few cases of biventricular thrombi undergoing VA-ECMO as bridge to transplantation have been reported. A few cases have reported of the use of VA-ECMO as a bridge to transplant, with total artificial heart serving as an alternative bridge [7].

The exact etiology of heart failure and biventricular thrombus formation remains unclear in this case as no hereditary or acquired causes of hypercoagulability or heart failure were revealed through our extensive testing. We hypothesize that due to unclear reasons (alcohol-indued cardiomyopathy cannot be ruled out) the patient developed biventricular failure and subsequent biventricular thrombus formation as a complication. It is plausible that thrombus fragments embolized to the coronary arteries, contributing to the significant myocardial infarction observed on explanted heart pathology [8].

The presence of massive biventricular thrombi significantly limited traditional treatment options for heart failure. Thrombectomy with subsequent LV assist device implantation is a common approach in patients with isolated LV thrombus and LV dysfunction [9]. However, our patient's biventricular dysfunction and the potential for RV failure following thrombectomy made him not an ideal candidate for LV assist device placement.

VA-ECMO as a bridge to OHT emerged as a viable option. While uncommonly used for biventricular thrombi, VA-ECMO offered hemodynamic support and potentially prevented systemic embolization of the thrombi due to increased afterload generated by VA-ECMO. In our case the patient already suffered from occlusion of the right common femoral artery and multiple PE complicated with lung infarcts, therefore guarding against development of further thromboembolism was paramount.

The successful heart transplantation in this case highlights the potential of VA-ECMO as a bridge to transplantation for patients with biventricular thrombi and end-stage cardiomyopathy. While this case report offers valuable insights, further research is necessary to establish the optimal management strategy for patients with this complex condition.

## Conclusion

This case report presents a complex scenario of a patient with acute heart failure complicated by massive biventricular thrombi, ultimately requiring heart transplantation. The successful utilization of VA-ECMO as a bridge to transplantation underscores the importance of a multidisciplinary approach in managing such critically ill patients.

The successful outcome emphasizes the importance of early recognition, aggressive management, and consideration of advanced therapies such as VA-ECMO and heart transplantation in this patient population**.**

## Consent statement

The patient provided consent before the case documentation began. However, all personal information has been excluded.

## Declaration of competing interest

The authors have no disclosures.
